# Supplementary material for: Decline of COPD exacerbations in clinical trials over two decades – a systematic review and meta-regression
Source: Respir Res. 2019 Aug 16;20:186. doi: 10.1186/s12931-019-1163-2 (PMC6697937; doi:10.1186/s12931-019-1163-2)
Supplement: Supplementary file 1 — Table S1. The list of references ordered by publication year, and alphabetically within each year. Publication numbers correspond to numbers shown at the top of Fig. 2. (PDF 476 kb) [file 12931_2019_1163_MOESM1_ESM.pdf]

Table A1: List of references ordered by publication year, and alphabetically within each year. Publication numbers shown in the left columns allow to identify the publications in the corresponding panel of Figure 2 (indicated by the tick marks at the top of each plot).

index			study
all	true	ics	
1		1	Boyd et al. (1997). An evaluation of salmeterol in the treatment of chronic obstructive pulmonary disease (COPD). *European Respiratory Journal*; 10(4):815-821.
2	1		Collet et al. (1997). Effects of an immunostimulating agent on acute exacerbations and hospitalizations in patients with chronic obstructive pulmonary disease. *American Journal of Respiratory and Critical Care Medicine*; 156(6):1719-1724.
3		2	Mahler et al. (1999). Efficacy of salmeterol xinafoate in the treatment of COPD. *Chest*; 115(4):957-965.
4	2		Burge et al. (2000). Randomised, double blind, placebo controlled study of fluticasone propionate in patients with moderate to severe chronic obstructive pulmonary disease: the ISOLDE trial. *BMJ*; 320(7245):1279-1303.
5		3	Chapman et al. (2002). The addition of salmeterol 50µg bid to anticholinergic treatment in patients with {COPD}: a randomized, placebo controlled trial. *Canadian Respiratory Journal*; 9(3):178-185.
6		4	Donohue et al. (2002). A 6-month, placebo-controlled study comparing lung function and health status changes in COPD patients treated with Tiotropium or Salmeterol. *Chest*; 122(1):47-55.
7		5	Rossi et al. (2002). Comparison of the efficacy, tolerability and safety of formoterol dry powder and oral, slow-release theophylline in the treatment of COPD. *Chest*; 121(4):1058-1069.
8	3		Calverley et al. (2003a). Combined salmeterol and fluticasone in the treatment of chronic obstructive pulmonary disease: a randomized controlled trial. *The Lancet*; 361(9356):449-456.
9	4		Calverley et al. (2003b). Maintenance therapy with budesonide and formoterol in chronic obstructive pulmonary disease. *European Respiratory Journal*; 22(6):912-919.
10	5		Celli et al. (2003). Symptoms are an important outcome in chronic obstructive pulmonary disease clinical trials: results of a 3-month comparative study using the Breathlessness, Cough and Sputum Scale (BCSS). *Respiratory Medicine*; 97:S35-S43.
11		6	Hiller et al. (2003). Long-term use of Viozan (sibenadet HCl) in patients with chronic obstructive pulmonary disease: results of a 1-year study. *Respiratory Medicine*; 97:S45-S52.
12	6		Szafranski et al. (2003). Efficacy and safety of budesonide/formoterol in the management of chronic obstructive pulmonary disease. *European Respiratory Journal*; 21(1):74-81.
13		7	Campbell et al. (2005). Formoterol for maintenance and as-needed treatment of chronic obstructive pulmonary disease. *Respiratory Medicine*; 99(12)1511-1520.
14	7		Rabe et al. (2005). Roflumilast - an oral anti-inflammatory treatment for chronic obstructive pulmonary disease: a randomized controlled trial. *The Lancet*; 366(9485)563-571.
15		8	Beeh et al. (2006). Wirksamkeit von Tiotropiumbromid (Spiriva) bei verschiedenen

			Schweregraden der chronisch-obstruktiven Lungenerkrankung (COPD). *Pneumologie*; 60(6):341-346.
16		9	Dusser et al. (2006). The effect of tiotropium on exacerbations and airflow in patients with COPD. *European Respiratory Journal*; 27(3):547-555.
17	8		Paggiaro et al. (2006). Multicentre randomized placebo-controlled trial of inhaled fluticasone propionate in patients with chronic obstructive pulmonary disease. *The Lancet*; 351(9195):773-780.
18	9		Rennard et al. (2006). Cilomilast for COPD: results of a 6-month, placebo-controlled study of a potent, selective inhibitor of phosphodiesterase 4. *Chest*; 129(1):56-66.
19		10	Zhou et al. (2006). Positive benefits of theophylline in a randomized, double-blind, parallel-group, placebo-controlled study of low-dose, slow-release theophylline in the treatment of COPD for 1 year. *Respirology*; 11(5):603-610.
20		11	Baumgartner et al. (2007). Nebulized arformeterol in patients with COPD: a 12-week, multicenter, randomized, double-blind, double-dummy, placebo- and active-controlled trial. *Clinical Therapeutics*; 29(2):261-278.
21	10		Calverley et al. (2007a). Effect of 1-year treatment with roflumilast in severe chronic obstructive pulmonary disease. *American Journal of Respiratory and Critical Care Medicine*; 176(2):154-161.
22		12	Calverley et al. (2007b). Salmeterol and fluticasone propionate and survival in chronic obstructive pulmonary disease. *The New England Journal of Medicine*; 356(8):775-789.
23	11		Zheng et al. (2007). The efficacy and safety of combination salmeterol (50 µg) / fluticasone propionate (500 µg) inhalation twice daily via accuhaler in Chinese patients with COPD. *Chest*; 132(6):1756-1763.
24		13	Ambrosino et al. (2008). Tiotropium and exercise training in COPD patients: effects on dyspnea and exercise tolerance *International Journal of Chronic Obstructive Pulmonary Disease*; 3(4):771-780.
25	12		Johansson et al. (2008). Bronchodilator efficacy of tiotropium in patients with mild to moderate COPD. *Primary Care Respiratory Journal*; 17(3):169-175.
26	13		Tashkin et al. (2008b). Efficacy and safety of budesonide and formoterol in one pressurized metered-dose inhaler in patients with moderate to very severe chronic obstructive pulmonary disease: Results of a 6-month clinical trial. *Drugs*; 68(14):1975-2000.
27		14	Tonnel et al. (2008). Effect of tiotropium on health-related quality of life as a primary efficacy endpoint in COPD. *International Journal of Chronic Obstructive Pulmonary Disease*; 3(2):301-310.
28		15	Dahl et al. (2010). Efficacy of a new once-daily long-acting inhaled beta-agonist indacaterol versus twice-daily formoterol in COPD. *Thorax*; 65(6):473-479.
29	14		Bogdan et al. (2011). Efficacy and safety of inhaled formoterol 4.5 and 9 µg twice daily in Japanese and European COPD patients: Phase III study results. *BMC Pulmonary Medicine*; 11:51.
30		16	Chapman et al. (2011). Long-term safety and efficacy of Indacaterol, a long-acting beta-agonist, in subjects with COPD. A randomized, placebo-controlled study. *Chest*; 140(1):68-75.
31		17	DUrzo et al. (2011) Efficacy and safety of once-daily NVA237 in patients with moderate-to-severe COPD: the GLOW1 trial. *Respiratory Medicine*; 12:156.
32		18	Jones et al. (2011a). Efficacy and safety of once-daily aclidinium in chronic obstructive pulmonary disease. *Respiratory Research*; 12:55.

33		19	Jones et al. (2011b). Efficacy and safety of once-daily aclidinium in chronic obstructive pulmonary disease. *Respiratory Research*; 12:55.
34	15		Lee et al. (2011). Roflumilast in asian patients with COPD: A randomized placebo-controlled trial. *Respirology*; 16(8):1249-1257.
35	16		Troosters et al. (2011). A 24 week, randomized, double-blind, placebo-controlled, multicenter study to evaluate the efficacy and safety of 18~mcg of tiotropium inhalation capsules administered by handihaler once-daily plus prn albuterol (salbutamol) vs. placebo plus prn albuterol (salbutamol) in chronic obstructive pulmonary disease subjects naive to maintenance therapy. *Clinical Study Report Synopsis, Boehringer-Ingelheim*, Protocol A4471008.
36	17		Doherty et al. (2012). Effects of mometasone furoate/formoterol fumarate fixed-dose combination formulation on chronic obstructive pulmonary disease (COPD): results from a 52-week Phase III trial in subjects with moderate-to-very severe COPD. *International Journal of COPD*; 7:57-71.
37		20	Jones et al. (2012). Efficacy and safety of twice-daily aclidinium bromide in COPD patients: the ATTAIN study. *European Respiratory Journal*; 40(4):830-836.
38		21	Kerwin et al. (2012). Efficacy and safety of NVA237 versus placebo and tiotropium in patients with COPD: the GLOW2 study. *European Respiratory Journal*; 40(5):1106-1114.
39	18		Tashkin et al. (2012). Efficacy and safety characteristics of mometasone furoate/formoterol fumarate fixed-dose combination in subjects with moderate to very severe COPD: findings from pooled analysis of two randomized, 52-week placebo-controlled trials. *International Journal of COPD*; 7:73-86.
40	19		Abrahams et al. (2013). Safety and efficacy of the once-daily anticholinergic BEA2180 compared with tiotropium in patients with COPD.<br>*Respiratory Medicine*; 107(6):854-862.
41		22	Bateman et al. (2013). Dual bronchodilation with QVA149 versus single bronchodilator therapy: the SHINE study. *European Respiratory Journal*; 42(6):1484-1494.
42		23	Donohue et al. (2013). Efficacy and safety of once-daily umeclidinium/vilanterol 62.5/25 mcg in COPD. *Respiratory Medicine*; 107(10):1538-1546.
43		24	Rennard et al. (2013). ACCORD COPD II: A randomized clinical trial to evaluate the 12-week efficacy and safety of twice-daily aclidinium bromide in chronic obstructive pulmonary disease patients. *Clinical Druc Investigation*; 33(12):893-904.
44		25	Donohue et al. (2014b). Safety and tolerability of once-daily umeclidinium/vilanterol 125/25 mcg and umeclidinium 125 mcg in patients with chronic obstructive pulmonary disease: results from a 52-week, randomized, double-blind, placebo-controlled study. *Respiratory Research*; 15:78.
45		26	D'Urzo et al. (2014).Efficacy and safety of fixed-dose combinations of aclidinium bromide/formoterol fumarate: the 24-week, randomized, placebo-controlled AUGMENT COPD study. *Respiratory Research*; 15:123.
46		27	Singh et al. (2014b). Efficacy and safety of aclidinium bromide/formoterol fumarate fixed-dose combinations compared with individual components and placebo in patients with COPD (ACLIFORM-COPD): a multicentre, randomised study. *BMC Pulmonary Medicine*; 14:178.
47		28	Trivedi et al. (2014). Umeclidinium in patients with COPD: a randomised, placebo-controlled study. *European Respiratory Journal*; 43(1):72-81.
48		29	Braido et al. (2015). Sub-lingual administration of a polyvalent mechanical bacterial lysate (PMBL) in patients with moderate, severe, or very severe chronic

			obstructive pulmonary disease (COPD) according to the GOLD spirometric classification: A multicentre, double-blind, randomised, controlled, phase IV study (AIACE study: Advanced Immunological Approach in COPD Exacerbation). *Pulmonary Pharmacology & Therapeutics*; 33:75-80.
49		30	Lee et al. (2015). Efficacy and safety of aclidinium bromide in patients with COPD: A phase 3 randomized clinical trial in a Korean population. *Respirology*; 20(8):1222-1228.
50		31	Wang et al. (2015). Efficacy and safety of once-daily glycopyrronium in predominantly Chinese patients with moderate-to-severe chronic obstructive pulmonary disease: the GLOW7 study. *International Journal of COPD*; 10:57-68.
51	20		Zheng et al. (2015a). Efficacy and safety of fluticasone furoate/vilanterol (50/25 mcg; 100/25 mcg; 200/25 mcg) in Asian patients with chronic obstructive pulmonary disease: a randomized placebo-controlled trial. *Current Medical Research and Opinion*; 31(6):1191-12000.
52		32	Zheng et al. (2015b). Efficacy and safety of once-daily inhaled umeclidinium/vilanterol in Asian patients with COPD: results from a randomized, placebo-controlled study. *International Journal of COPD*; 10:1753-1767.
53	21		Bhatt et al. (2017). A randomized trial of once-daily fluticasone furoate/vilanterol or vilanterol versus placebo to determine effects on arterial stiffness in COPD. *International Journal of COPD*; 12:351-365.
54		33	DUrzo et al. (2017). A randomised double-blind, placebo-controlled, long-term extension study of the efficacy, safety and tolerability of fixed-dose combinations of aclidinium/formoterol or monotherapy in the treatment of chronic obstructive pulmonary disease. *Respiratory Medicine*; 125:39-48.
55		34	Maltais et al. (2018). Effect of 12 weeks of once-daily tiotropium/ olodaterol on exercise endurance during constant work-rate cycling and endurance shuttle walking in chronic obstructive pulmonary disease. *Therapeutic Advances in Respiratory Disease*; 12:1-13.
